# Lysophosphatidic Acid Signaling in Cancer

**DOI:** 10.3390/cancers12123791

**Published:** 2020-12-16

**Authors:** David N. Brindley

**Affiliations:** 1 Department of Biochemistry, University of Alberta, Edmonton, AB T6G 2S2, Canada; david.brindley@ualberta.ca; Tel.: +1-780-492-2078; Fax: +1-780-492-3383; 2 Cancer Research Institute of Northern Alberta, University of Alberta, Edmonton, AB T6G 2S2, Canada

This Special Issue aims to highlight the impact of discoveries made over the last 25 years on the role of autotaxin (ATX) and lysophosphatidic acid (lysophosphatidate, LPA) signaling in tumor growth, metastasis and the treatment of cancers by chemotherapy, radiotherapy and immunotherapy. These studies were greatly facilitated by the identification of six G-protein-coupled receptors for LPA and the discovery that LPA signaling is terminated by the degradation of extracellular LPA by a family of three lipid phosphate phosphatases (LPP1–3). LPP1 also attenuates signaling downstream of the activation of LPA and protease-activated receptors. The Special Issue consists of nine reviews and four original research articles.

As a result of pre-clinical research on ATX/LPA signaling, there are now a variety of therapeutic agents that inhibit LPA synthesis by ATX and LPA signaling through its various receptors or increase LPA degradation. So far, these potential therapeutics have not been used to decrease the adverse effects of LPA signaling in the management of cancer patients. This is a real need because it is now clear that LPA signaling is an important component of tumor-induced inflammation, which is an essential promoter of cancer progression, metastasis and fibrosis. Furthermore, inflammation and increased LPA signaling leads to immune evasion, which helps to protect cancer cells from destruction by the body’s immune system. We are now at the exciting point of being able to target LPA signaling as a novel paradigm for improving existing cancer treatments. This becomes feasible because ATX inhibitors and LPA receptor antagonists have already entered clinical trials for the treatment of idiopathic pulmonary fibrosis.

Benesch et al. [1] develop ideas that ATX and LPA signaling could provide novel adjuvant targets for overcoming the loss of efficacy in chemotherapy and radiotherapy and also attenuate adverse therapeutic side-effects, including radiation-induced fibrosis. Such agents and future compounds, which target ATX/LPA signaling, could find new applications for cancer treatments. The review [1] discusses how ATX and LPA participate in a feed-forward cycle of inflammation that is part of natural protection in wound healing. When healing is complete, inflammation resolves and lower concentrations of inflammatory cytokines no longer support high levels of ATX secretion. However, in chronic inflammatory conditions, the increases in ATX/LPA signaling and the survival and proliferative signals from LPA become maladaptive, leading to a variety of pathologies, including metastatic cancers and fibrosis. Benesch et al. [1] focus their discussions on breast cancer, where locally produced ATX is supplied mainly by adipose tissue that becomes inflamed by cytokines released by adjacent breast tumors. This situation contrasts with thyroid cancers, melanomas and glioblastomas, where the cancer cells produce ATX directly in response to inflammatory cytokines.

Meng et al. [2] studied the effects of ionizing radiation that was focused on breast fat pads of normal and tumor-bearing mice. This mimics the situation where the post-operative breast of cancer patients is subjected to multiple daily fractions (e.g., 16 fractions at 2.65 Gy) of radiation after lumpectomy. One fraction of 7.5 Gy of X-radiation in the mouse model produced an early effect of increasing plasma ATX activity. This probably resulted from DNA damage in irradiated adipocytes, which are a major site of ATX secretion. The observed decrease in plasma adiponectin concentrations can be explained by the direct action of LPA signaling on adipocytes. Decreases in plasma adiponectin concentrations indicate adverse effects on metabolism. Three daily fractions of 7.5 Gy augmented the tumor-induced increases in plasma ATX activity and decreased adiponectin levels in the associated mammary fat pad. There were increased expressions of several inflammatory mediators in the breast tumors and adjacent mammary fat pad after three fractions of radiation, which were accompanied by increased infiltration of CD45+ leukocytes [2]. This wound-healing response to radiation therapy (RT)-induced damage could decrease the efficacy of further fractions of RT. The following paper by Meng et al. [3] showed that administration of the anti-inflammatory agent dexamethasone (DEX) during RT attenuated the increases in ATX activity in the plasma and mammary adipose tissue and also the increase in LPA_1_ receptor levels in adipose tissue after 48 h. DEX treatment during five daily fractions of 7.5 Gy also attenuated fibrotic changes associated with vasculitis in blood vessels where ATX was mainly localized in the breast fat pad and underlying lungs at 7 weeks after the RT. Dexamethasone decreased macrophage inflammatory protein 2-alpha (CXCL2), active transforming growth factor-β1, connective tissue growth factor and nuclear factor erythroid 2–related factor 2 (Nrf2) at 7 weeks in adipose tissue of irradiated mice [3]. These combined studies indicate that increased ATX production and LPA signaling contribute to RT-induced breast inflammation, vasculitis and fibrosis.

The review by Kaffe et al. [4] discusses different aspects of how the interactions of ATX, LPA and LPA receptors control the progression of liver fibrosis and cancer. They focus on metabolic, viral and cholestatic liver disorders and how this affects the progression to liver cancer in human patients and mouse models. In particular, the role of ATX/LPA in non-alcoholic fatty liver disease and its progression to liver cancer is discussed. Because adipose tissue accounts for the largest amount of ATX production in the body, many studies have implicated LPA in adipose tissue metabolism and inflammation, liver steatosis, insulin resistance, glucose intolerance and lipogenesis. Equally, ATX and LPA are crucial in the development of fibrotic diseases, and hepatocellular carcinoma usually develops when there is also liver fibrosis (cirrhosis). Kaffe et al. [4] propose that attenuating the progression of fibrosis could help to delay the progression to hepatocellular carcinoma. Signaling by ATX and LPA appears to be an attractive therapeutic target because of the important role of LPA in the progression of both liver fibrosis and liver cancer.

Kostadinova et al. [5] also focused their review on the role of ATX in liver disease and especially in the setting of chronic hepatitis C virus (HCV) and HCV/human immunodeficient virus (HIV) infection. Circulating ATX is elevated in people with liver disease and it is proposed that this is a consequence of impaired liver clearance secondary to fibrotic liver disease. The authors identified plasma ATX as being associated with parameters of systemic immune activation during HCV and HCV/HIV infections. There is a partial normalization of ATX levels within months of starting direct-acting antiviral HCV therapy that is interferon-free. This is consistent with a non-fibrotic liver disease contribution to elevated ATX levels, or HCV-mediated hepatocyte activation. Kostadinova et al. [5] then discuss the relationships between ATX/LPA signaling and systemic immune activation and consider this in the context of HCV infection, age, immune health, liver health and hepatocellular carcinoma.

Peyruchaud et al. [6] focus their review on the role of inflammation in increasing ATX expression, particularly in the osteoarticular compartment, where this leads to increased bone erosion. The biological actions of ATX are mediated by LPA and this also requires protection of LPA from degradation by the action of lipid phosphate phosphatases. They propose that this can be achieved by interaction of LPA through docking to a carrier protein. ATX could, itself, act as a docking molecule for LPA. Binding of ATX to the cell surface through interaction with molecules such as integrins and heparan sulfate proteoglycans could facilitate a rapid route of delivering active LPA to its cell surface receptors [6]. This new paradigm of ATX/LPA signaling could have an important impact on how we could treat metastatic cancers and inflammatory bone diseases.

The review by Salgado-Polo and Perrakis [7] discusses a related theme from structural studies that have been performed on ATX. ATX has a bimetallic nucleophilic catalytic site, a substrate-binding (orthosteric) hydrophobic pocket that accommodates the lipid alkyl chain and an allosteric tunnel that can accommodate various steroids and LPA. The authors discuss what is known about ATX-mediated catalysis, crucially in light of allosteric regulation. They also present the known functions of ATX that are independent of catalysis. These include binding to cell-surface integrins and proteoglycans, which could have possible roles in delivering LPA to its receptors. The crystal structures of ATX bound to various inhibitors show that the inhibitors can be separated into four classes that are established based on binding to the orthosteric and/or the allosteric site. Type I inhibitors prevent LPA production from lysophosphatidylcholine, but they do not affect independent functions of the allosteric site. By contrast, Type IV inhibitors can also abolish any functionality of the allosteric site, expelling bound LPA, which could be essential for the clinical success of the Type IV inhibitors [7]. The authors point out that endothelial differentiation gene (EDG) and non-EDG LPA receptors have different characteristics in their binding pockets. This could indicate that the delivery of LPA to the different LPA receptors through the tunnel in ATX could be receptor-dependent. The binding differences are discussed in terms of the different mechanistic effects that can differentially modulate the response of the ATX–LPA signaling axis effects, including the use of the inhibitors for clinical applications, including cancer therapy.

The review by Lee et al. [8] concentrates on one of the hallmarks of cancer, namely the ability of cancer cells to modulate and escape immune detection and eradication. Despite information about the role of LPA in regulating immune functions and inflammation, its role in tumor immunity has received little attention. The authors discuss recent studies that show how the ATX/LPA signaling axis could enable cancer cells to evade immune detection and eradication. This area of research is extremely important because at least 20 immunotherapies have been approved by the U.S. Food and Drug Administration (FDA) to treat cancers of the skin, lung, bladder, kidney, stomach, liver, prostate and breast, multiple myeloma, leukemia and Hodgkin’s diseases. Immunotherapy can be highly effective. However, it is estimated that of the 44% of cancer patients who are eligible for current checkpoint inhibitors, only 13% of patients will respond well to these therapies. Hence, there is a need to understand the mechanisms that regulate immune responses in cancer patients to develop improved treatment strategies. For example, inhibition of LPA5 receptors in CD8^+^ T cells could boost immune surveillance against cancer cells and impede metastasis [8]. The evidence provided in this review emphasizes the potential that drugs that target ATX and LPA signaling could be used as adjuvants to improve patient outcomes from immunotherapy in addition to effects on chemotherapy and RT.

The review by Dr. Yun emphasizes that the intestinal epithelium interacts dynamically with the immune system so that it can maintain an effective barrier function to protect the host while performing physiological roles in the absorption of nutrients, electrolytes, water and minerals [9]. LPA and its receptors in gut physiology have been progressively appreciated as being important for the maintenance of the epithelial barrier so that it is able to provide the first line of defense against the milieu of potentially pathogenic stimuli. This review focuses on how LPA-mediated signaling can become dysfunctional and contribute to inflammation in the gastrointestinal tract. This has other long-term consequences because inflammatory bowel disease can progress to colorectal cancer.

Dr. Xu addresses the complexity of LPA metabolism, its different receptors and its signaling systems [10]. It is proposed that detecting signatures and networks, rather than individual genes and expressions of proteins and lipids from individual patients will be needed to develop effective treatments for highly heterogenic diseases such as cancers. There is a need for multi-modular integrative approaches consolidating large amounts of data from gene expression profiling, next-generation sequencing, -omics studies, prognostic/predictive modeling and functional studies for cancer. Dr. Xu highlighted one study that used a systems biology approach to identify novel sphingolipid–LPA immune checkpoints and networks underlying tumor immune heterogeneity and disease outcomes in ovarian cancers [11]. This type of study could hold great promise for delivering novel stratifying and targeting strategies. As in other articles in this series, Dr. Xu recommends that targeting LPA signaling as monotherapy for cancers is unlikely to be effective. A major challenge will be to discover combinations of therapeutic modalities that will be effective in the treatment of different cancers in individual patients.

Dr. Murph emphasizes that the functions of miRNAs in normal tissues can become aberrant in malignant cells depending on the roles of the miRNAs as tumor suppressors or oncogenic factors [12]. This review discusses the current status of miRNA regulation in the context of LPA production by ATX and LPA signaling through its receptors. Some miRNAs increase in response to LPA stimulation, including miR-21, miR-30c-2-3p and miR-122. Other miRNAs inhibit the translation of LPA receptors, such as miR-15b, miR-23a and miR200c. miRNAs, such as miR-146 and miR-21, regulate proteins that are downstream of LPA signaling. It is concluded that more miRNAs, which are uncharacterized at present, will be identified as regulators of LPA signaling. RNA-based therapeutics have entered the clinic, with enormous potential to provide new approaches to precision medicine.

Harper et al. [13] investigated the responses of the ATX–LPA–LPP axis to hypoxia, which is a common characteristic of advanced solid tumors and a potent driver of tumor invasion and metastasis. Although there is evidence for the involvement of ATX and LPA receptors in cancer cell invasion promoted by hypoxia in the tumor microenvironment, the transcriptional and/or spatiotemporal control of this process remains unexplored. The authors studied whether hypoxia promotes cell invasion by altering LPA production through ATX and its degradation by LPP1 and LPP3. While LPP3 expression was downregulated by hypoxia, ATX and LPP1 were asymmetrically redistributed to the leading edge and to the trailing edge of cancer cells, respectively [13]. This was associated with the opposing functions of ATX and the LPPs in cell invasion and it controls the generation of an LPA gradient that drives cellular invasion and migration in the hypoxic zones of tumors.

The article by Yagi et al. [14] discusses discrepancies in the literature where LPA concentrations from ovarian cancer patients have been reported. Practical advice is provided on how to collect plasma samples to minimize changes in LPA concentrations that can occur during processing and storage.

The collection of articles in this Special Issue on LPA signaling and cancers will hopefully stimulate more research in this area and the eventual introduction of new therapeutics that will attenuate the adverse effects of signaling through the ATX–LPA inflammatory axis. This should improve patient outcomes from chemotherapy, RT and immunotherapy and decrease fibrosis induced by cancers and RT.

## References

[1] Benesch M.G.K., Tang X., Brindley D.N. (2020). Autotaxin and Breast Cancer: Towards Overcoming Treatment Barriers and Sequelae. Cancers.

[2] Meng G., Wuest M., Tang X., Dufour J., Zhao Y., Curtis J.M., McMullen T.P.W., Murray D., Wuest F., Brindley D.N. (2019). Repeated Fractions of X-Radiation to the Breast Fat Pads of Mice Augment Activation of the Autotaxin-Lysophosphatidate-Inflammatory Cycle. Cancers.

[3] Meng G., Tang X., Yang Z., Zhao Y., Curtis J.M., McMullen T.P.W., Brindley D.N. (2019). Dexamethasone decreases the autotaxin-lysophosphatidate-inflammatory axis in adipose tissue: Implications for the metabolic syndrome and breast cancer. FASEB J..

[4] Kaffe E., Magkrioti C., Aidinis V. (2019). Deregulated Lysophosphatidic Acid Metabolism and Signaling in Liver Cancer. Cancers.

[5] Kostadinova L., Shive C.L., Anthony D.D. (2019). Elevated Autotaxin and LPA Levels During Chronic Viral Hepatitis and Hepatocellular Carcinoma Associate with Systemic Immune Activation. Cancers.

[6] Peyruchaud O., Saier L., Leblanc R. (2019). Autotaxin Implication in Cancer Metastasis and Autoimunne Disorders: Functional Implication of Binding Autotaxin to the Cell Surface. Cancers.

[7] Salgado-Polo F., Perrakis A. (2019). The Structural Binding Mode of the Four Autotaxin Inhibitor Types that Differentially Affect Catalytic and Non-Catalytic Functions. Cancers.

[8] Lee S.C., Dacheux M.A., Norman D.D., Balazs L., Torres R.M., Augelli-Szafran C.E., Tigyi G.J. (2020). Regulation of Tumor Immunity by Lysophosphatidic Acid. Cancers.

[9] Yun C.C. (2019). Lysophosphatidic Acid and Autotaxin-associated Effects on the Initiation and Progression of Colorectal Cancer. Cancers.

[10] Xu Y. (2019). Targeting Lysophosphatidic Acid in Cancer: The Issues in Moving from Bench to Bedside. Cancers.

[11] Meshcheryakova A., Svoboda M., Jaritz M., Mungenast F., Salzmann M., Pils D., Cacsire Castillo-Tong D., Hager G., Wolf A., Braicu E.I. (2019). Interrelations of Sphingolipid and Lysophosphatidate Signaling with Immune System in Ovarian Cancer. Comput. Struct. Biotechnol. J..

[12] Murph M.M. (2019). MicroRNA Regulation of the Autotaxin-Lysophosphatidic Acid Signaling Axis. Cancers.

[13] Harper K., Brochu-Gaudreau K., Saucier C., Dubois C.M. (2019). Hypoxia Downregulates LPP3 and Promotes the Spatial Segregation of ATX and LPP1 During Cancer Cell Invasion. Cancers.

[14] Yagi T., Shoaib M., Kuschner C., Nishikimi M., Becker L.B., Lee A.T., Kim J. (2019). Challenges and Inconsistencies in Using Lysophosphatidic Acid as a Biomarker for Ovarian Cancer. Cancers.

